# PD-1 silencing impairs the anti-tumor function of chimeric antigen receptor modified T cells by inhibiting proliferation activity

**DOI:** 10.1186/s40425-019-0685-y

**Published:** 2019-08-07

**Authors:** Jianshu Wei, Can Luo, Yao Wang, Yelei Guo, Hanren Dai, Chuan Tong, Dongdong Ti, Zhiqiang Wu, Weidong Han

**Affiliations:** Department of Bio-therapeutic, Department of Molecular & Immunology, Chinese PLA General Hospital, No. 28 Fuxing Road, Beijing, 100853 China

**Keywords:** PD-1 blockade, Chimeric antigen receptor modified T cells, T cell proliferation, T cell differentiation, Persistence

## Abstract

**Background:**

Blocking programmed death-1 (PD-1) is considered to be a promising strategy to improve T cell function, and this is being explored in many ongoing clinical trials. In fact, our knowledge about PD-1 is primarily based on the results of short-term experiments or observations, but how long-lasting PD-1 blockade can affect T cell function remains unclear.

**Methods:**

We planned to use shRNA-based gene knockdown technology to mimic long-lasting PD-1 blockade. We constructed PD-1 steadily blocked chimeric antigen receptor modified T (CAR-T) cells, and with these cells we can clearly study the effects of PD-1 knockdown on T cell function. The anti-tumor function, proliferation ability and differentiation status of PD-1 silenced CAR-T cells were studied by in vitro and animal experiments.

**Results:**

According to short-term in vitro results, it was reconfirmed that the resistance to programmed death-ligand 1 (PD-L1)-mediated immunosuppression could be enhanced by PD-1 blockade. However, better anti-tumor function was not presented by PD-1 blocked CAR-T cells in vitro or in vivo experiments. It was found that PD-1 knockdownmight impair the anti-tumor potential of CAR-T cells because it inhibited T cells’ proliferation activity. In addition, we observed that PD-1 blockade would accelerate T cells’ early differentiation and prevent effector T cells from differentiating into effect memory T cells, and this might be the reason for the limited proliferation of PD-1 silenced CAR-T cells.

**Conclusion:**

These results suggest that PD-1 might play an important role in maintaining the proper proliferation and differentiation of T cells, and PD-1 silencing would impair T cells’ anti-tumor function by inhibiting their proliferation activity.

**Electronic supplementary material:**

The online version of this article (10.1186/s40425-019-0685-y) contains supplementary material, which is available to authorized users.

## Background

Chimeric antigen receptor modified T (CAR-T) cells exhibit potent antitumor activity against hematological malignancies [1–4]. However, the translation of this success to solid tumors is still gloomy [5]. In the treatment of solid tumors, CAR-T therapy is faced with enormous difficulties, such as the immunosuppressive milieu [6, 7]. In the establishment of the suppressive milieu, programmed death-1 (PD-1)/ programmed death-ligand 1 (PD-L1) axis is thought to play a key role [6, 8, 9].

As an inhibitory receptor, PD-1 inhibits T cells activity by engaging with its ligands [10, 11]. It has been widely confirmed that PD-1 blocking antibodies could help cytotoxic T lymphocytes (CTL) resist immune suppression and enhance anti-tumor functions [12–14]. And PD-1 antibodies were also reportedly able to rescue CAR-T cells from exhaustion and senescence [15, 16]. In addition to antibodies, intrinsic PD-1 blocking by genetic modification was also proved to be effective [17, 18]. Therefore, PD-1 blockade is considered to be a promising method to improve CAR-T cell function and is explored in many ongoing clinical trials.

Although this concept has solid theoretical foundation, so far few clinical outcomes clearly prove its authenticity. This dilemma inspired us to re-cognize PD-1 blockade.

In fact, the conclusion that PD-1 blockade can improve T cell function is mostly based on the results of short-term experiments or observations; however, the PD-1 blocking in clinical practice is usually long-lasting. This means that there is a cognitive gap between our knowledge and clinical practice, and the missing link is that we still don’t know how long-lasting PD-1 blockade will affect T cell function.

Actually, some studies have suggested that long-lasting PD-1 blockade might induce negative feedback regulations. It has been reported that persistently blocking PD-1 (both with antibodies and with genetic modification) would up-regulate T cell immunoglobulin and mucin-domain containing-3 (TIM-3) and lymphocyte activation gene-3 (LAG-3) [19, 20], which forms an important mechanism to resist PD-1 blockade. In a fraction of patients, a novel pattern of hyperprogressive disease (HPD) induced by anti-PD-1 treatment was observed [21, 22]. It has also been reported that PD-1 knockout would promote exhaustion of CD8-positive T cells, and PD-1 was believed to play an important role in preventing T cells from overstimulation and senescence [23]. Although these studies demonstrated the possibility of the negative regulation, the effects of long-lasting PD-1 blockade on T cell functions have not been systematically evaluated, which we think is very necessary.

To this end, we constructed dual-promoter lentivirus vectors which allowed us to simultaneously express the PD-1 targeting short hairpin RNA (shRNA) and CAR molecule (ZsGreen followed). With this approach, we could clearly analyze every single CAR-T cell, whose PD-1 is persistently blocked. We observed that persistent PD-1 silencing would significantly impair the anti-tumor potential of CAR-T cells, especially in long-term tumor killing or at lower effector cell to target cell (E:T) ratios. The proliferation activities, both cytokines dependent and CAR-mediated activation dependent, were found to be attenuated dramatically by PD-1 knockdown, which we believed was the main cause of CAR-T cells’ impaired anti-tumor potential. In addition, it was suggested that the early-differentiation of CAR-T cells was accelerated by PD-1 knockdown, which is generally considered to be detrimental to T cell proliferation and persistence.

These findings illustrate that PD-1 signaling might not always be unfavorable for T cell functions. Moreover, it is essential for preventing over-rapid differentiation and maintaining normal proliferative activity. This work would help us understand the long-lasting PD-1 blockade more comprehensively, and it might have important implications for the clinical application of PD-1 blockade therapy.

## Methods

### Dual-promoter lentivirus vector construction and viral production

The dual-promoter lentivirus vector, pLVX-ShRNA-IRES-ZsGreen from Biowit Biotech, was used as the basal framework for construction. We inserted the CAR sequences into the downstream of EF1a promoter and replaced the IRES sequence with the T2A sequence. Six different PD-1 targeting shRNA sequences were inserted into the downstream of U6 promoter respectively. A widely used scramble shRNA sequence was constructed into the vector in the same manner as control. Its sequence is as follows:

- Sense: 5′-GATCCCGCGCGATAGCGCTAATAATTTCTCGAGAAATTATTAGCGCTATCGCGCTTTTTTGGAAA-3′.
- Antisense: 5′-CGCGTTTCCAAAAAAGCGCGATAGCGCTAATAATTTCTCGAGAAATTATTAGCGCTATCGCGCGG-3′.

After validation by sequencing, these expressing plasmids were co-transfected with three packaging plasmids (pLP1, pLP2 and pLP/VSVG) into 293 T packaging cell lines to produce lentivirus.

### Cell lines

A549 adenocarcinomic human alveolar basal epithelial cells (ATCC) were infected with lentivirus to express human CD19 and ZsGreen simultaneously, and purified CD19 positive A549 cells were obtained by fluorescence activated cell sorting (FACS).

These cells were then infected with the pLenti-CMV-luc2-IRES-Puro virus to express firefly luciferase. And the firefly luciferase stably expressing cells were established by puromycin selection. The Raji human Burkitt’s lymphoma cells (ATCC) were engineered similarly to express luciferase.

### Preparation of CAR-T cells

CAR-T cells were generated from donors’ peripheral blood mononuclear cells (PBMCs). For T cells activation, 1 μg/ml anti-CD3 monoclonal antibody (OKT3, Takara) was pre-coated overnight at 4 °C and 50 ng/ml anti-CD28 monocolonal antibody (CD28.2, Biolegend) was added into the medium. The PBMCs were activated for 2 days before infection. Infection was carried out by centrifugation at 850 g in a 24-well plate at 31 °C for 2 hours, and an agent called Envirus™-LV (Engreen Biosystem) was applied to promote the infection efficiency. After infection, the CAR-T cells were cultured in GT-T551 medium (Takara) with 0.5% fetal bovine serum (FBS, Gibco) and 300 U/ml recombinant human IL-2 (rhIL-2, PeproTech).

In another culture protocol, 10 ng/ml recombinant human IL-7 (rhIL-7, PeproTech), 10 ng/ml recombinant human IL-15 (rhIL-15, PeproTech) and 10 ng/ml recombinant human IL-21 (rhIL-21, PeproTech) were used instead of IL-2.

### Quantitative real-time PCR

The mRNA was reversed into cDNA using Transcriptor First Strand cDNA Synthesis Kit (Roche). Quantitative Real-Time PCR (qRT-PCR) was performed with FastStart Universal Real-Time PCR Master Mix (Roche) on Applied Biosystems 7500 systems. The primers were designed and synthesized by Life Technologies. The comparative Ct was normalized to the β-actin housekeeping gene as follows: Δ^Ct^ (sample) = Ct (PD-1) – Ct (β-actin). Then, the relative expression folds compared with control were calculated as follows: 2 -ΔΔCt = 2^-(ΔCt [sample] – ΔCt [control]).

To test the intratumoral CAR copy numbers, the DNA from tumors was directly used as templates for qRT-PCR. The primers targeting a 153 base pair fragment containing portions of the CD8 a chain and adjacent CD137 chain wereused. To calculate the relative copy numbers folds, the sample with the highest Δ^Ct^ in the S4-CART19 group on the seventh day was used as the control.

The following are the sequences of the primers used to detect PD-1 and CAR:

- PD1-F-1:AGATCAAAGAGAGCCTGCGG,
- PD1-R-1:CTCCTATTGTCCCTCGTGCG;
- PD1-F-2:GTGTCACACAACTGCCCAAC,
- PD1-R-2:CTGCCCTTCTCTCTGTCACC;
- PD1-F-3:TGCAGCTTCTCCAACACATC,
- PD1-R-3:CACGCTCATGTGGAAGTCAC;
- CAR-F: GGTCCTTCTCCTGTCACTGGTT,
- CAR-R:TCTTCTTCTTCTGGAAATCGGCAG.

### Western blotting (WB) and immunofluorescence staining

Seventy-two hours after infection, the 293 T cells were collected for WB or immunofluorescence staining. The antibody targeting CD3-ζchain (ab119827, Abcam) was used to detect the CAR molecule.

After infection, ZsGreen positive Jurkat human T lymphocyte cells were sorted by FACS. After expansion, antibody targeting PD-1 (ab52587, Abcam) was used to detect the expression of PD-1.

### Flow cytometry

All operations were performed in accordance with the manufacturer’s recommended protocols. For detecting intracellular antigens, Foxp3/Transcription Factor Staining Buffer Set (00–5523-00, eBioscience) was used for fixation and perforation. The antibodies used in the text are as follows: mouse Fab fragment (115–065-072, Jackson ImmunoResearch), biotin (554061, BD pharmigen), PD-1 (329906, 329908 and 329938, Biolegend), PD-L1 (393610, Biolegend), CD3 (300308 and 300326, Biolegend), TIM-3 (345012, Biolegend), LAG-3 (369312, Biolegend), CD45RO (304210, Biolegend), CD62L (304840, Biolegend), CD107a (328620, Biolegend), IFN-γ (502512, Biolegend) and Ki67 (350540, Biolegend). The 7-AAD (17501, AAT Bioquest) was used to detect apoptosis. DRAQ5 (65–0880-92, eBioscience) was used for live cells cell cycle assay.

Flow cytometry was performed on BD’s Calibur II platform and the datas were analyzed by FlowJo software. For cell cycle analysis, the Modifit LT software was used.

### T cell function analysis

To test the Ki67 and PD-1 expression upon target cells stimulation, 1 × 10^5^ CAR-T cells and target cells were co-cultured for 12 h in 96-well plate, and then the cells were subjected to flow cytometry analysis.

To test the expression of CD107a and IFN-γ, 1 × 10^5^ CAR-T cells and target cells were co-cultured for 4 h in 96-well plate. During the co-culture, Golgi inhibitors monensin (420701, Biolegend) and Brefeldin A (420601, Biolegend) were added. For CD107a analysis, the CD107a antibody was added into the medium at the beginning of the co-culture.

To test the released inflammatory factors, 1 × 10^5^ purified CAR-T cells and target cells were co-cultured for 24 h in 96-well plate. Subsequently, the culture supernatant was used for multi-factor flow assay. Pre-defined panels of LEGENDplex (Biolegend) were used, and the LEGENDplex v8.0 software was used for analysis.

To test the in vitro proliferation, 0.4 × 10^4^ purified CAR-T cells and 0.4 × 10^5^ Raji-luc cells were co-cultured for 72 h in 96-well plate. The absolute numbers of T cells were calculated by the following formula: total cell count × proportion of CD3 positive T cells.

To test the lysis of target cells, purified CAR-T cells were co-cultured with target cells at different E:T ratios in 96-well plate. 100 μl 2 × D-Luciferin solution (300 μg/ml) were added into each well, and the signals were measured after 2–5 min by Varioskan™ LUX (Thermo Fisher). The lysis was calculated by the following formula: 1-([value of sample]-[value of negative control])∕([value of positive control]-[value of negative control]).

### Mouse models

We established intraperitoneally injected Raji-luc lymphoma and subcutaneously implanted A549–19luc solid tumor models, in which female NOD-Prkdc^scid^-Il2rg^null^ mice (NPG/Vst, VITALSTAR) aged 4 to 6 weeks were used. The volume of cells per injection was 100 μl. CAR-T cells were resuspended in PBS and injected through the tail vein. Tumor burdens were quantified by bioluminescence imaging (BLI) on NightOwl II (LB 983, Berthold) platform, BLI data was analyzed using indiGO software (Berthold); BLI signal was reported as average flux (photons per second∕area [mm^2^]). All animals were anesthetized with isoflurane gas.

For tracing the T cells in peripheral blood, approximately 200 μl blood was taken through the canthus for subsequent flow analysis. To analyze the copy numbers of intratumoral CAR-T cells, the tumor masses were obtained by excision and fixed in formalin for subsequent qRT-PCR experiments.

### Statistics

Data were analyzed using Prism v7.0 (GraphPad Software) and SPSS Statistics 24 (IBM). Survival curves were analyzed using the log-rank test. Statistical significance was defined as *P* < 0.05.

### Study approval

All animal experiments have been approved by the Institutional Animal Care and Use Committee (IACUC) of Chinese PLA General Hospital (PLAGH), and all the procedures were performed in accordance with the guidelines of IACUC of PLAGH.

## Results

### Preparation of PD-1 knockdown CART-19 cells

**Fig. 1:**
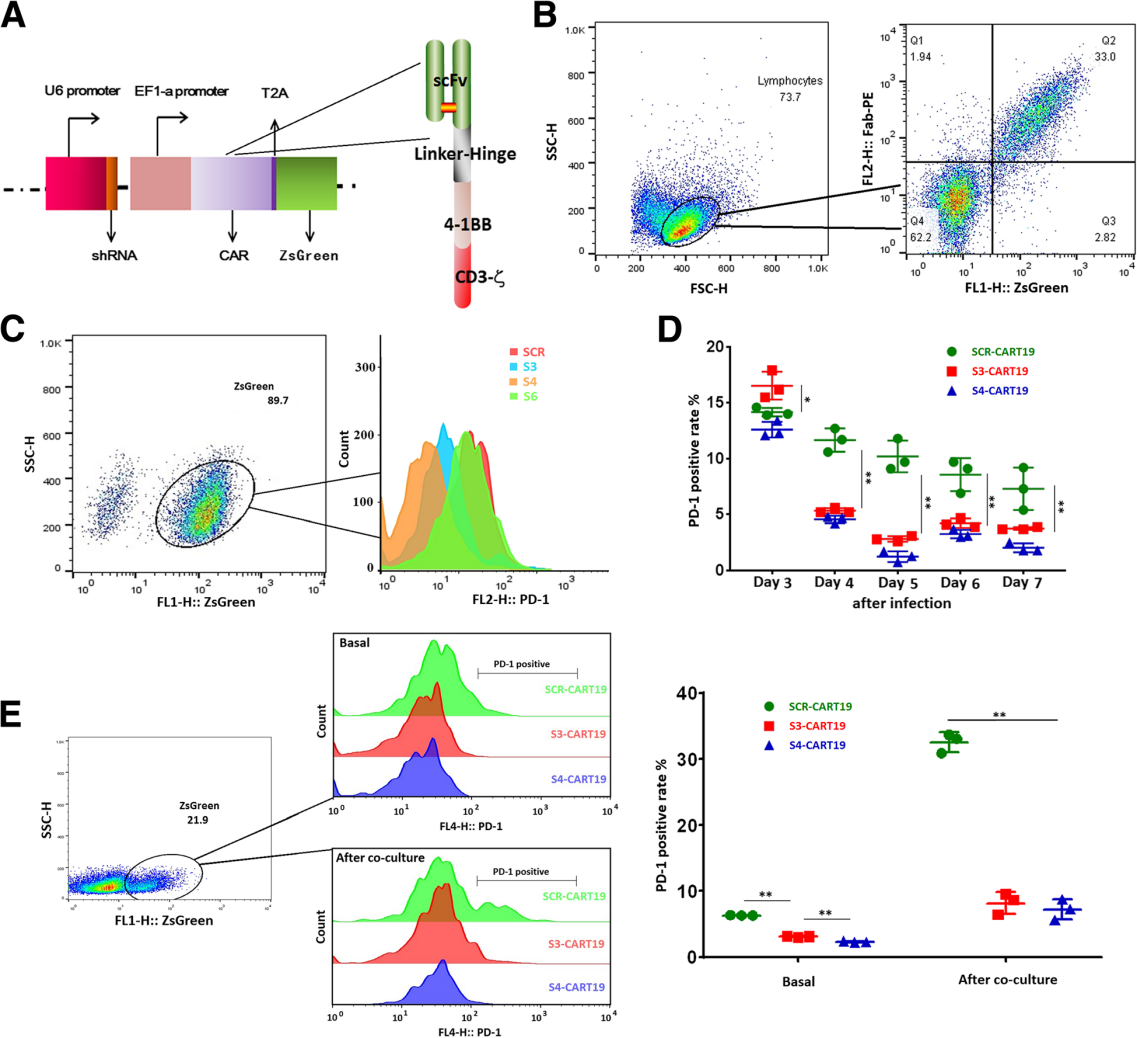
Preparation of PD-1 stably blocked CART19 cells. **a** Schematic representation of dual promoter lentivirus vectors and CAR structure. **b** Anti-mouse Fab antibody and PE conjucted second antibody were used to detect CAR. A strict one-to-one correspondence between the expression of CAR and ZsGreen was demonstrated. **c** In ZsGreen positive sorted Jurkat cells, S3 and S4 presented effective PD-1 knockdown efficiency, while S6 showed no significant effect. **d** From the fourth day after infection, S3 and S4 showed significant PD-1 knockdown efficiency in CAR-T cells. CAR-T **e** the expressions of PD-1 in different CAR-T cell populations (9 days after virus infection) were tested before and after 24 h co-culture with Raji cells. During co-culture, S3 and S4 significantly decreased the expression of PD-1. 0.01 < *P < 0.05; **P < 0.01. Statistical significance was determined using the ANOVA method for multiple comparisons. Data represent the mean ± SEM of triplicates and are representative of at least 3 independent experiments or are plotted as individual points

We constructed dual-promoter vectors those could express PD-1 targeting shRNA and CAR simultaneously, to which a ZsGreen motif was linked by a T2A motif. The expression of shRNA was driven by U6 promoter, and elongation factor 1-alpha (EF1-α) was used to initiate the expression of CD19-targeting CAR (CAR19) (Fig. 1a).

The expression of CAR19 was confirmed by WB and immunofluorescence (Additional file 1: Figure S1A and B). As shown in Fig. 1b, a strict one-to-one correspondence between the expression of CAR and ZsGreen was presented, which allowed us to track and purify CAR-T cells by ZsGreen. The infection efficiency was between 15 and 25% on the third day after infection, and no significant difference between different constructs was demonstrated.

Six different PD-1 targeting shRNA sequences were synthesized to screen for valid ones, and a scramble sequence (SCR) was used as control. The PD-1 silencing efficiency was firstly analyzed in Jurkat cells by qRT-PCR, WB and flow cytometry (Additional file 1: Figure S1C, D and Fig. 1c) to exclude invalid shRNA sequences. Finally, we screened out two valid shRNA sequences, shRNA-3 (S3) and shRNA-4 (S4).

The functions of S3 and S4 were further confirmed in T cells. The expression of PD-1 was not significantly inhibited by S3 or S4 until the fourth day after lentivirus infection. On the seventh day of culture, which was the fifth day after viral infection, the PD-1 positive rates in S3 and S4 modified CART-19 (S3-CART19 and S4-CART19) cells decreased by about 72 and 88% respectively compared with that in the SCR modified CART-19 (SCR-CART19) cells (Fig. 1d).

In addition, we confirmed that the expression of PD-1 in SCR-CART19 cells would be significantly up-regulated by target cell-induced immune response (TCIIR) after 24 h co-culture with Raji cells (Additional file 1: Figure S1F). And the up-regulations could be effectively inhibited by S3 and S4 (Fig. 1e). The in vivo CAR and PD-1 expression in different CAR-T cells were also detected 7 days after CAR-T infusion (Additional file 1: Figure S3A). Most of the CD3-positive T cells expressed CAR molecule, and S3 and S4 could effectively inhibit the expression of PD-1.

Due to the more pronounced PD-1 silencing efficiency, S4 was chosen for the following functional tests.

### PD-1 knockdown did not enhance the cytotoxicity of CAR-T cells

**Fig. 2:**
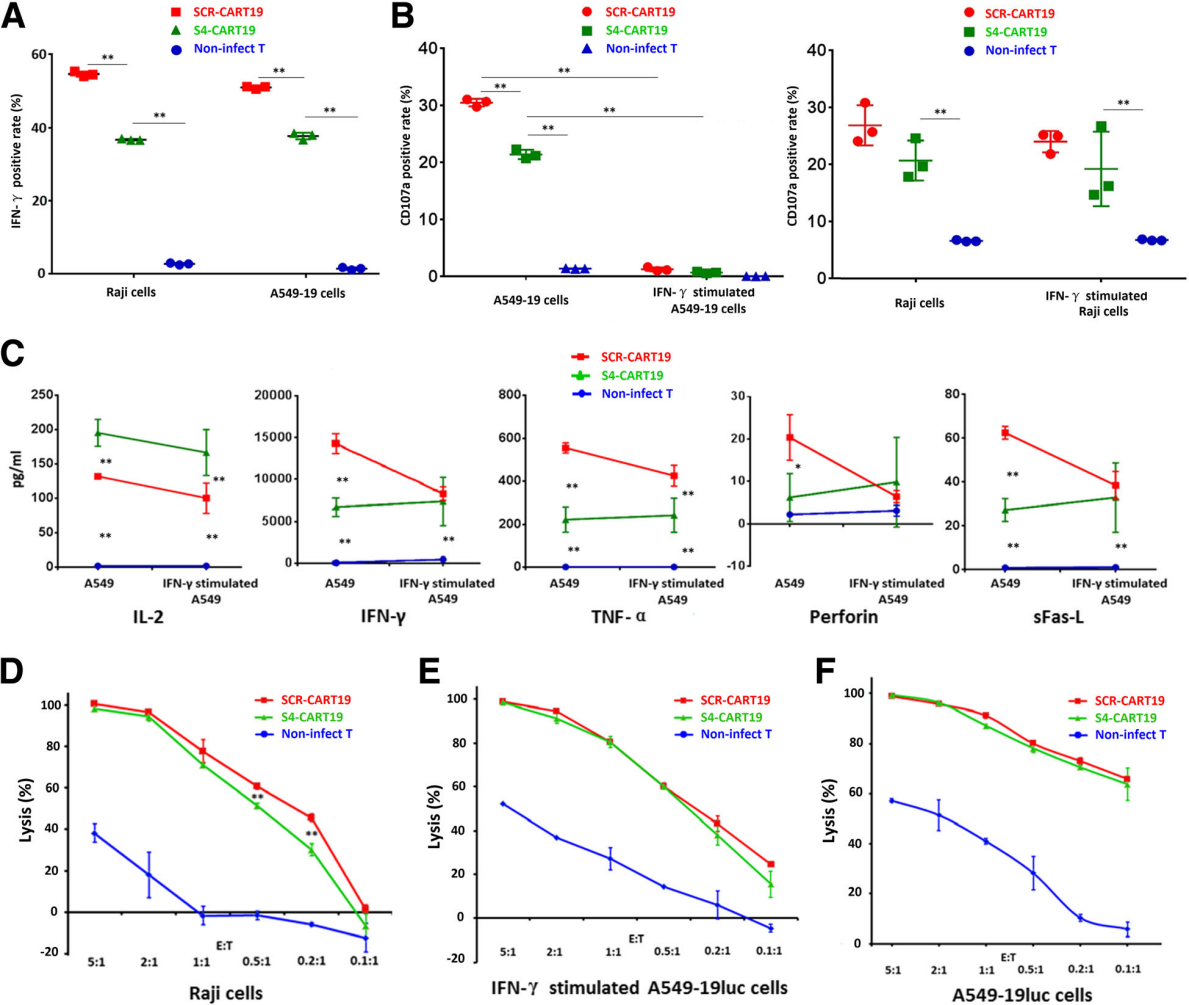
PD-1 knockdown did not enhance the cytotoxicity of CAR-T cells, but instead impaired it. **a** A549–19 and Raji cells both stimulated the expression of IFN-γ in CAR-T cells, and the expression of IFN-γ was impaired by PD-1 knockdown. **b** SCR-CART19 cells presented a higher positive rate of TCIIR induced CD107a expression. High expression of PD-L1 almost completely inhibited the CD107a expression. **c** PD-1 blockade promoted the secretion of IL-2 but not the other tested cytokines, however, better resistance to PD-L1-mediated immunosuppression was clearly indicated. **d-f** Lysis of Raji-luc cells (**d**), IFN-γ stimulated A549–19luc cells (**e**) and A549–19luc cells (**f**) at different E:T ratios were measured by luminescence. PD-1 knockdown impaired the lysis ability on Raji cells at low E:T ratios. **P < 0.01. Statistical significance was determined using the ANOVA method for multiple comparisons. Data represent the mean ± SEM of triplicates and are representative of at least 3 independent experiments or are plotted as individual points

To evaluate the TCIIR potential, the expression of IFN-γ and CD107a of CAR-T cells cultured for 7 days were detected after co-culture with Raji and CD19 positive A549 cells (A549–19). As shown in Fig. 2a and b, co-culture induced a higher positive rates of IFN-γand CD107a in SCR-CART19 cells than that in S4-CART19 cells, and the strong expression of PD-L1 significantly inhibited CAR-T cells CD107a expression (Fig. 2b and Additional file 1: Figure S2). This indicated that PD-1 knockdown might impair CAR-T cells TCIIR in this short-time co-culture experiment. Moreover, the residual PD-1 could still exert significant inhibitory effect, and this was further confirmed by CD107a expression test with A549–19 cells expressing moderate level of PD-L1 (Additional file 1: Figure S5D).

Inflammatory cytokines secretion is another important factor in determining anti-tumor activity. Multi-factor flow assay demonstrated that more IL-2 was secreted by S4-CART19 cells (cultured for 10 days). However, the secretions of the other tested factors were decreased by PD-1 knockdown. In this experiment, PD-1 knockdown significantly increased CAR-T cells resistance to PD-L1-mediated immunosuppression (Fig. 2c).

To test the cell lysis directly, Raji and A549–19 cells expressing firefly luciferase (Raji-luc and A549–19luc) were established. Purified CAR-T cells (cultured for 10 days) were co-cultured with target cells for 24 h. It was demonstrated that S4-CART19 and SCR-CART19 cells presented similar CAR-specific lysis of Raji-luc cells at higher E:T ratios. But at lower E:T ratios, 0.2:1 and 0.1:1, S4-CART19 cells was slightly, but with significance, less effective than SCR-CART19 cells (Fig. 2d). However, no significant difference in lysis of A549–19luc or IFN-γ-stimulated A549–19luc cells between SCR-CART19 and S4-CART19 was demonstrated (Fig. 2e and f). We speculated that this might be because the cytokines released by T cells during co-culture up-regulated the expression of PD-L1 in A549 cells, and this was proved true by the results presented in Additional file 1: Figure S5C.

Taken together, these results indicated that PD-1 knockdown did not enhance the cytotoxicity of CAR-T cells, but instead impaired it under certain conditions.

### Long-lasting PD-1 knockdown would impair the in vivo anti-tumor function of CAR-T cells

**Fig. 3:**
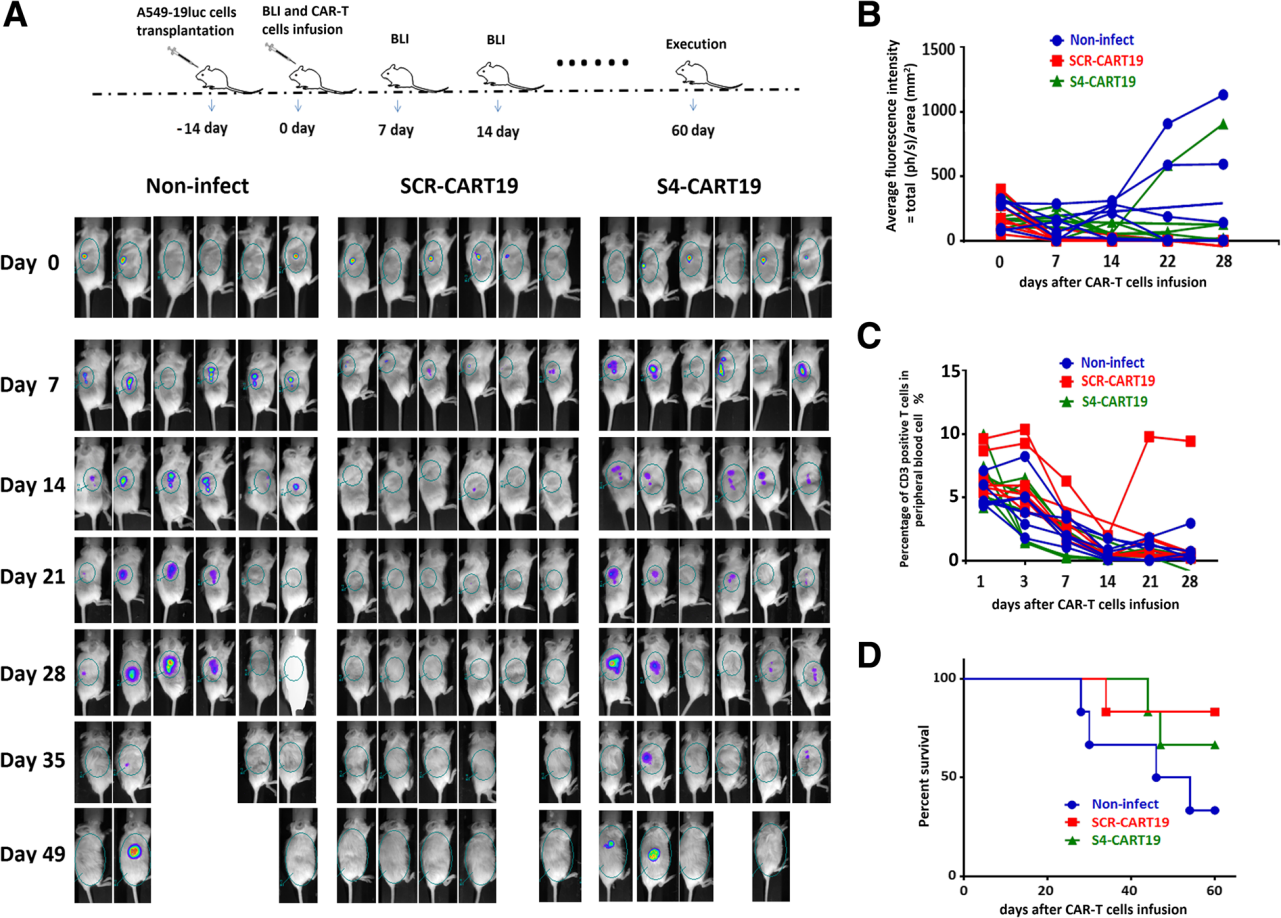
PD-1 blocked CAR-T cells demonstrated impaired anti-tumor effects in vivo. **a** A549–19luc xenograft model was established by subcutaneously injection of 5 × 10^6^ tumor cells per mouse. And then, the tumor bearing mice were treated with different CAR-T cells or non-infected T cells as control. S4-CART19 did not show better anti-tumor function than SCR-CART19. **b** Average fluorescence intensity in each mouse was measured to study the tumor burden, and the changes of tumor burden within 4 weeks after CAR-T reinfusion were presented here. SCR-CART19 inhibited the tumor growth more obviously. **c** The percentage of CD3 positive T cells was used to evaluate the number of CAR-T cells. And the data for each mouse within 4 weeks after CAR-T reinfusion indicated that the amplification of CAR-T was not obvious in this model. **d** Survival curves were analyzed using the log-rank test, and the statistical result did not prove that there was a significant difference between SCR-CART19 and S4-CART19 treated mice. Data are plotted as individual points

To evaluate the effect of long-lasting PD-1 knockdown on T cells, we conducted an in vivo experiment (Fig. 3a). In this xenograft model, 5 × 10^6^ A549–19luc cells were implanted subcutaneously 2 weeks before CAR-T infusion. Tumor burdens across groups were equalized by BLI before CAR-T treatment. For CAR-T treatment, 1 × 10^6^ sorted CAR-T cells cultured for 10 days were administered, and non-infected T cells were used as control. It was found that SCR-CART19 cells could rapidly eradicate tumors, and no tumor recurrence was detected during the following 2 months observation. Better anti-tumor function of S4-CART19 cells was not observed, on the contrary, the tumor clearance was significant slower and weaker (Fig. 3a, b and Additional file 1: Figure S3B). All the treated mice exhibited little T cells expansion, except for one receiving SCR-CART19 cells developed significant T cells amplification 3 weeks after CAR-T cells infusion (Fig. 3c and Additional file 1: Figure S3C). The survival statistics showed that the mice treated with S4-CART19 cells did not have prolonged survival compared with those treated with SCR-CART19 cells (Fig. 3d).

These results suggested that long-lasting PD-1 silencingmight impair the in vivo anti-tumor function of CAR-T cells.

### PD-1 knockdown impaired CAR-T cells in vitro proliferative potential

Proliferation is a key factor in determining CAR-T cells’ anti-tumor potential [24]. In previous in vivo experiments, the effect of PD-1 knockdown on T cell proliferation was not demonstrated due to the insufficient expansion of CAR-T cells. Therefore, we conducted more specific in vitro experiments to study the proliferative capacity of CAR-T cells.

**Fig. 4:**
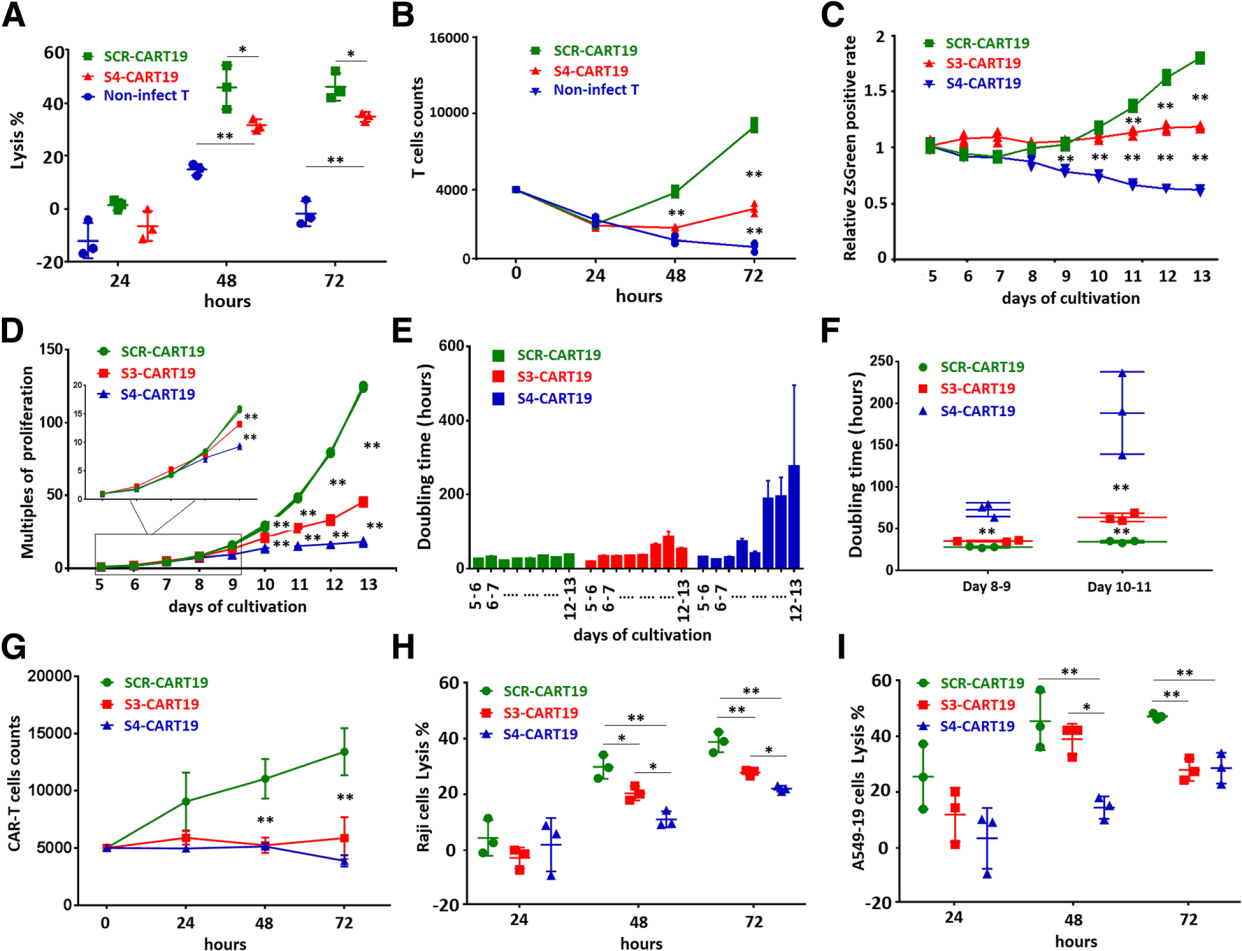
PD-1 knockdown impaired CAR-T cells in vitro proliferative potential. **a** 0.4 × 10^4^ purified CAR-T cells were co-cultured with Raji-luc cells at the E:T ratio of 01:1 for 72 h. Lysis of Raji-luc cells at different time were measured by luminescence, and S4-CART19 presented restrained lysis ability than SCR-CART19. **b** The absolute numbers of T cells were recorded daily to evaluate the in vitro proliferation potential which was driven by target cells stimulation, and the proliferation was significantly impaired by PD-1 knockdown. **c** The ZsGreen positive rates in different T cell populations were continuously recorded which were divided by the mean positive rates on the fifth day in each group to get the relative value of the positive rate. **d** The absolute numbers of CAR-T cell were continuously recorded, and the PD-1 silenced CAR-T cells presented impaired proliferative potential. **e** The daily doubling time of CAR-T cells were calculated, and the proliferation of S3-CART19 and S4-CART19 slowed down significantly with the prolongation of cultivation, compared with SCR-CART19. **f** Compared with SCR-CART19, the doubling time of S4-CART19 and S3-CART19 increased significantly from day 8 to day 9 and from day 10 to day 11, respectively. **g** 0.5 × 10^4^ purified CAR-T cells were co-cultured with A549–19 cells at the E:T ratio of 01:1 for 72 h. The absolute numbers of T cells were recorded daily, and SCR-CART19 proliferated more significantly than S3-CART19 and S4-CART19. **h** 0.5 × 10^5^ Raji-luc cells were co-cultured with 0.5 × 10^4^ purified CAR-T cells for 72 h, and SCR-CART19 presented higher lysis ability than S3-CART19 and S4-CART19. **i** 0.5 × 10^5^ A549–19luc cells were co-cultured with 0.25 × 10^4^ purified CAR-T cells for 72 h, and SCR-CART19 presented higher lysis ability than S3-CART19 and S4-CART19.CAR-T 0.01 < *P < 0.05; **P < 0.01. Statistical significance was determined using the ANOVA method for multiple comparisons. Data represent the mean ± SEM of triplicates and are representative of at least 3 independent experiments or are presented individually

First, 0.4 × 10^4^ purified CAR-T cells cultured for 10 days were co-cultured with Raji-luc cells at a low E:T ratio (0.1:1) for 3 days. The tumor lysis by S4-CART19 cells was found to be significantly impaired compared with SCR-CART19 cells (Fig. 4a). Meanwhile, the amplification times of S4-CART19 cells was only about one-third of that of SCR-CART19 cells (Fig. 4b), indicating that the amplification driven by TCIIR was impaired.

Next, we tested the expression of ki67 in CAR-T cells to further elucidate the proliferative potential. It was confirmed that the ki67 positive rate of S4-CART19 cells was lower than that of SCR-CART19 cells not only after but also before TCIIR (Additional file 1: Figure S4A). This suggested that CAR-T cells proliferation driven by cytokines might be also impaired by PD-1 silencing. This conclusion was conflicting to the widely accepted view that PD-1 receptor was detrimental to T cells proliferation [25]. We doubted whether the shRNA4 sequence mistakenly targeted other genes involved in cell proliferation. To exclude this possibility, another PD-1 targeting shRNA sequence, S3, was tested to verify the results’ authenticity.

We examined the expression of ki67 in CAR-T cells those were cultured for fourteen days. The results showed that S3 could produce similar effects on CAR-T cells as S4, although the effect was a little weaker (Additional file 1: Figure S4B).

By continuously tracking the ZsGreen positive rates during cultivation, we further confirmed that the proliferations of S3-CART19 and S4-CART19 cells were both damped, but the attenuation in S3-CART19 cells was delayed and more moderate than S4-CART19 cells (Fig. 4c). By calculating the absolute number of CAR-T cells, it was found that on the ninth day of culture (day 7 after viral infection), the proliferation multiples of S3-CART19 and S4-CART19 began to be significantly lower than SCR-CART19 (Fig. 4d). The daily doubling time was also calculated, and the proliferation of PD-1 silenced CAR-T cells became slower and slower with the prolongation of cultivation (Fig. 4e). Compared with SCR-CART19, the obvious prolongation of doubling time in S4-CART19 and S3-CART19 first appeared on day 8 to day 9 and on day 10 to day 11, respectively (Fig. 4f). Cell cycle assays also demonstrated that the proliferative potential during cultivation was impaired by PD-1 silencing, and the effect of S3 was weaker than that of S4 (Additional file 1: Figure S4C and D).

The 7-AAD staining results indicated that the decrease of PD-1 silenced CAR-T cells in total population was not due to increased apoptosis (Additional file 1: Figure S5A). We detected several genes most likely to be mistargeted by S3 or S4, and the qRT-PCR data further confirmed the specificity of S3 and S4 (Additional file 1: Figure S6).

The proliferation of S3-CART19 and S4-CART19 driven by co-culture with A549–19 cells (PD-L1 would be significantly up-regulated) were also tested. 0.5 × 10^4^ purified CAR-T cells were co-cultured with A549–19 cells at the E:T ratio of 01:1 for 72 h. The absolute T cell numbers were recorded daily, and SCR-CART19 presented more significant proliferation than S3-CART19 and S4-CART19 (Fig. 4g). The 72 h lysis analysis (E:T at 0.1:1 for Raji-luc cells and E:T at 0.05:1 for A549–19luc cells) demonstrated that SCR-CART19 presented higher lysis ability than S3-CART19 and S4-CART19 (Fig. 4h and i).

Taken together, these results demonstrated that PD-1 silencing in CAR-T cells would impair their proliferative potential, as well as the authenticity of this finding.

### T cells’ differentiation kinetics was altered by PD-1 knockdown

**Fig. 5:**
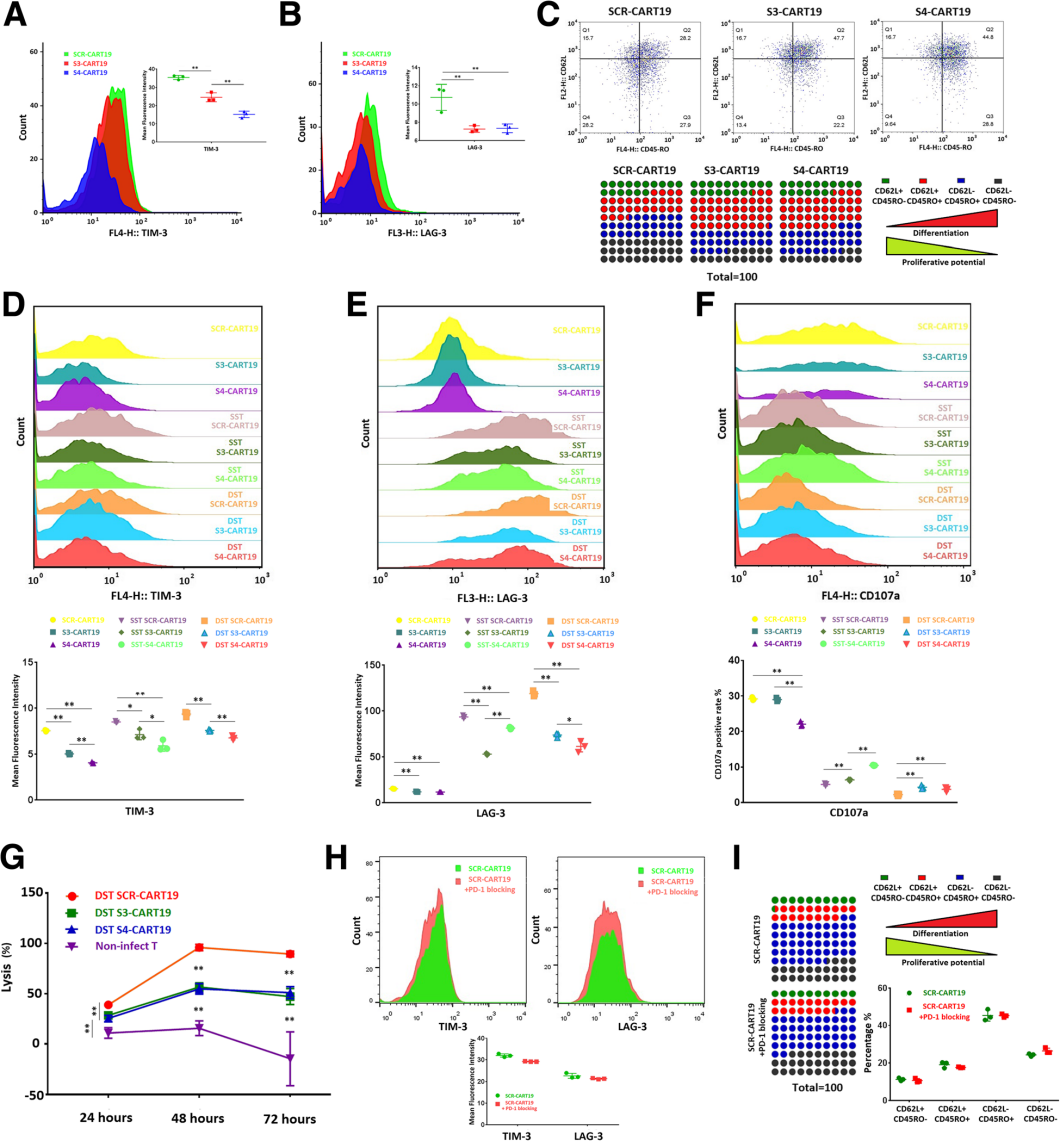
PD-1 knockdown altered T cells’ differentiation kinetics. Tim-3 and LAG-3 in CAR-T cells those were cultured for 13 days were examined by flow cytometry. The expressions of TIM-3 (**a**) and LAG-3 (**b**) in PD-1 silenced CAR-T cells were both lower than that in normal CAR-T cells. **c** The differentiation phenotypes of CD8 positive CAR-T cells cultured for 7 days were tested by flow cytometry. After SST or DST, the expression of TIM-3 (**d**) and LAG-3 (**e**) were tested. PD-1 silenced CAR-T cells expressed lower TIM-3 and LAG-3 than SCR-CART19 did. **f** After SST or DST, the TCIIR induced CD107a expressions were tested. Both SST and DST impaired CAR-T cells CD107a expression, which could be partially rescued by S3 and S4. **g** The lysis of Raji-luc cells at E:T = 1:5 were continuously monitored for 72 h, and DST SCR-CART19 still presented higher lysis ability than DST S3-CART19 and DST S4-CART19. **h** CAR-TThe expression of TIM-3 (left) and LAG-3 (right) and the differentiation phenotypes (**i**) were evaluated in SCR-CART19 cells those were cultured for 10 days. PD-1 blockade by antibodies did not present a similar effect on CAR-T cells as intrinsic PD-1 silencing. 0.01 < *P < 0.05; **P < 0.01. Statistical significance was determined using the ANOVA method for multiple comparisons. Data represent the mean ± SEM of triplicates and are representative of at least 3 independent experiments or are plotted as individual points

Differentiation status plays a decisive role in T cell proliferation. We assessed CAR-T cells’ senescence by detecting the expression of TIM3 and LAG3. It was found that PD-1 knockdown did not up-regulate the expression of TIM3 or LAG3, but decreased them. Meanwhile, this effect was more pronounced in S4-CART19 cells compared with S3-CART19 cells (Fig. 5a and b). During cultivation, T cells would gradually differentiate from early-differentiation status into late-differentiation status, and the proliferative capacity would also gradually decrease [26]. We labeled CD62L and CD45RO for CD8-positive T cells to analyze their differentiation status. Typically, CD62L^+^CD45RO^−^ T cells are considered to be naive T cells. As differentiation progresses, T cells would become CD62L^+^CD45RO^+^ central memory T cells and CD62L^−^CD45RO^+^ effector T cells. Although there are some different opinions about the status of CD62L^−^CD45RO^−^ T cells, we tend to believe that these cells are effector memory T cells derived from effector T cells.

As shown in Fig. 5c and Additional file 1: Figure S5E and F, during the cultivation, knockdown of PD-1 significantly accelerated CAR-T cells’ differentiation into CD62L^−^CD45RO^+^ T cells. In particular, the naive T cells in S3-CART19 and S4-CART19 faded rapidly. Meanwhile, PD-1 knockdown appeared to prevent further differentiation of CD62L^−^CD45RO^+^ T cells into CD62L^−^CD45RO^−^ T cells. By analyzing ZsGreen negative T cells (non-infected T cells) in the same cell populations (cultured for ten days), we excluded the possibility that different culture conditions caused this difference (Additional file 1: Figure S5B).

Next, the effect of PD-1 silencing on exhausted CAR-T cells was studied. Raji-luc cells were added at E:T = 1:3 on the 5th day of culture (single stimulation, SST) or on the 5th and 8th day of culture (double stimulation, DST) to induce T cell exhaustion. As shown in Fig. 5d, e and f, DST induced more significant exhaustion phenotype than SST, proofed by higher up-regulation of TIM-3 and LAG-3 and lower TCIIR induced CD107a expression. Of note, PD-1 knockdown seemed to retard T cell exhaustion during SST and DST. However, after DST, the antitumor functions of PD-1 knockdown CAR-T cells were still weaker than that of SCR-CART19 cells (Fig. 5g).

The effect of long-lasting PD-1 blockade by antibodies was also observed. We found that the antibodies barely altered the expressions of TIM-3 or LAG-3, differentiation kinetics and proliferative ability (Fig. 5h, i and Additional file 1: Figure S7). This suggested that simply blocking PD-L1/PD-1 interaction (T cells also express PD-L1, Additional file 1: Figure S1E) was quite different from intrinsic PD-1 silencing.

Taken together, we confirmed that PD-1 knockdown but not antibody-mediated blockade altered CAR-T cells’ differentiation kinetics.

### PD-1 knockdown impaired in vivo persistence and proliferation of CAR-T cells

**Fig. 6:**
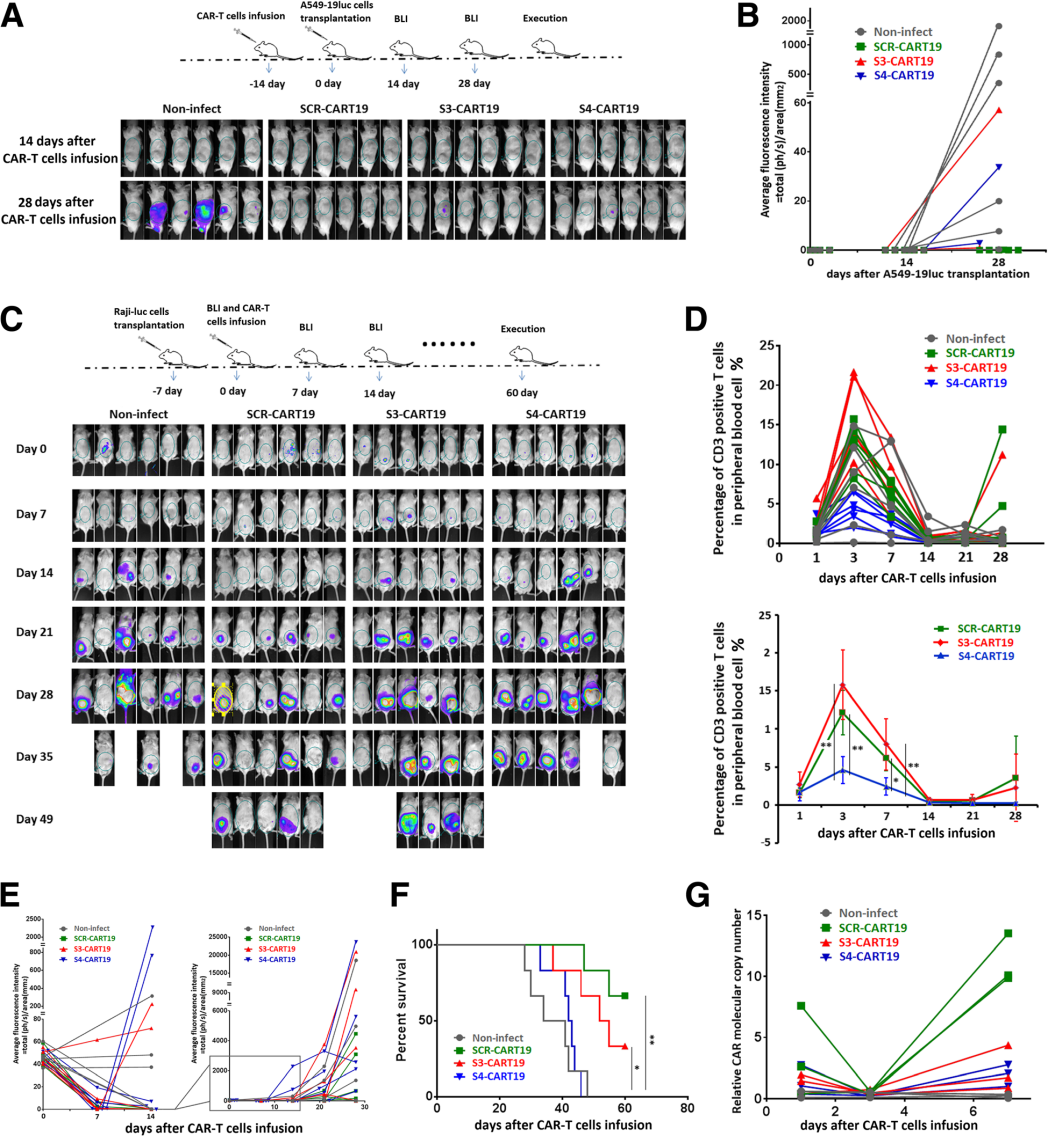
The in vivo persistence and proliferation of CAR-T cells were evaluated. (A and B) 1 × 10^6^ sorted CAR-T cells per mouse were administrated by intravenous injection. After 2 weeks of metabolism, the abilities to limit tumor growth of the infused CAR-T cells were assessed by re-challenge with A549–19luc cells. BLI images (**a**) and statistical data (**b**) both indicated that the residual SCR-CART19 cells limited tumor growth more significantly. **c** Raji-luc xenograft model was established by injection of 1 × 10^6^ tumor cells per mouse inoculated into abdominal cavity 1 week before the CAR-T treatment. And then, the tumor bearing mice were treated with different CAR-T cells or non-infected T cells as control. The tumor burdens were measured by bioluminescence imaging. **d** The percentage of CD3 positive T cells was used to evaluate the number of CAR-T cells. And the data for each mouse (up) and the mean value of each group (down) were presented here within 4 weeks after CAR-T reinfusion. **e** Average fluorescence intensity in each mouse was measured to study the tumor burden, and the changes in tumor burden within 4 weeks after CAR-T reinfusion were presented here. **f** Survival curves were analyzed using the log-rank test, and the percent survival for each group was presented. These results showed that the in vivo anti-tumor function and proliferation ability of CAR-T cells were impaired by PD-1 knockdown. **g** The copy numbers of tumor infiltrating CAR-T cells were examined by qRT-PCR to evaluate the intratumoral expansion. And PD-1 silenced CAR-T cells were shown to have impaired intratumoral proliferation. 0.01 < *P < 0.05; **P < 0.01. Statistical significance was determined using the ANOVA method for multiple comparisons. Data represent the mean ± SEM of triplicates and are representative of at least 3 independent experiments or are plotted as individual points

Persistence is another key factor determining CAR-T cells’ function [24]. To evaluate the persistence, we administered 1 × 10^6^ purified CAR-T cells or non-infected T cells per mouse. After 2 weeks of feeding, these mice were inoculated with 2 × 10^7^ A549–19luc cells subcutaneously. As shown in Fig. 6a, little tumor growth was observed in each group during the first 2 weeks after inoculation. During the next 2 weeks, tumors in control group grew rapidly. In comparison, the residual S3-CART19 and S4-CART19 cells limited tumor growth effectively, but the limitation was significantly less effective than that of SCR-CART19 cells (Fig. 6a and b). This suggested the persistence of CAR-T cells might be impaired by PD-1 knockdown.

In previous in vivo experiments, CAR-T cells did not show significant expansion, which might be due to the limited contacts between CAR-T cells and transplanted tumors. Therefore, we planned to use a hematological tumor model in which the contacts were sufficient to study the T cells proliferation. In this model, 1 × 10^6^ Raji-luc cells were inoculated 1 week before the CAR-T treatment (Fig. 6c). After equalization by BLI, 5 × 10^5^ CAR-T cells that were cultured for 10 days were given. As shown in Fig. 6d, the infused T cells reached a numerical peak on the third day after treatment (including the non-infected T cells) and then gradually fell back. In the fourth week after treatment, significant secondary amplifications of CAR-T cells were observed in two SCR-CART19 treated and one S3-CART19 treated mice. The statistical results showed that the expansion of S4-CART19 cells was significantly lower than that of SCR-CART19 and S3-CART19 cells. Tumor cells could be rapidly erased in almost all the CAR-T treated mice during the first week of treatment. During the second week, tumor clearance persisted in the SCR-CAR-T group, meanwhile, significant tumor growth was observed in both S3-CART19 and S4-CART19 groups. During the following 2 weeks, tumor burdens increased rapidly in all groups, but SCR-CART19 cells presented better anti-tumor function than S3-CART19 and S4-CART19 cells (Fig. 6c and e). And the survival statistics was consistent this view (Fig. 6f).

Considering that PD-L1-mediated immunosuppression could be partially rescued by PD-1 blockade, different amplification potential might be presented by solid tumor infiltrating CAR-T cells. To study the intratumoral expansion, 1 × 10^7^ A549–19luc cells were implanted subcutaneously. Three weeks later, the tumors, of which the diameters were about 1 cm, were equalized by BLI. And then 1 × 10^6^ purified CAR-T cells which were cultured for 10 days were given. After infusion, the qRT-PCR results showed that infiltrating CAR-T cells showed a significant proliferation on day 7 after a decline on day 3. The mean copy number of SCR-CART19 cells was significantly higher, about five times, than that of the S3-CART19 and S4-CART19 cells (Fig. 6g). This suggested that the intratumoral amplification was also impaired significantly by PD-1 silencing.

### The effects of PD-1 knockdown were prevalent in different culture conditions and CAR-T systems

Limited proliferation potential is one of the hallmarks of T cell exhaustion and this could be promoted or rescued by many factors [27].

**Fig. 7:**
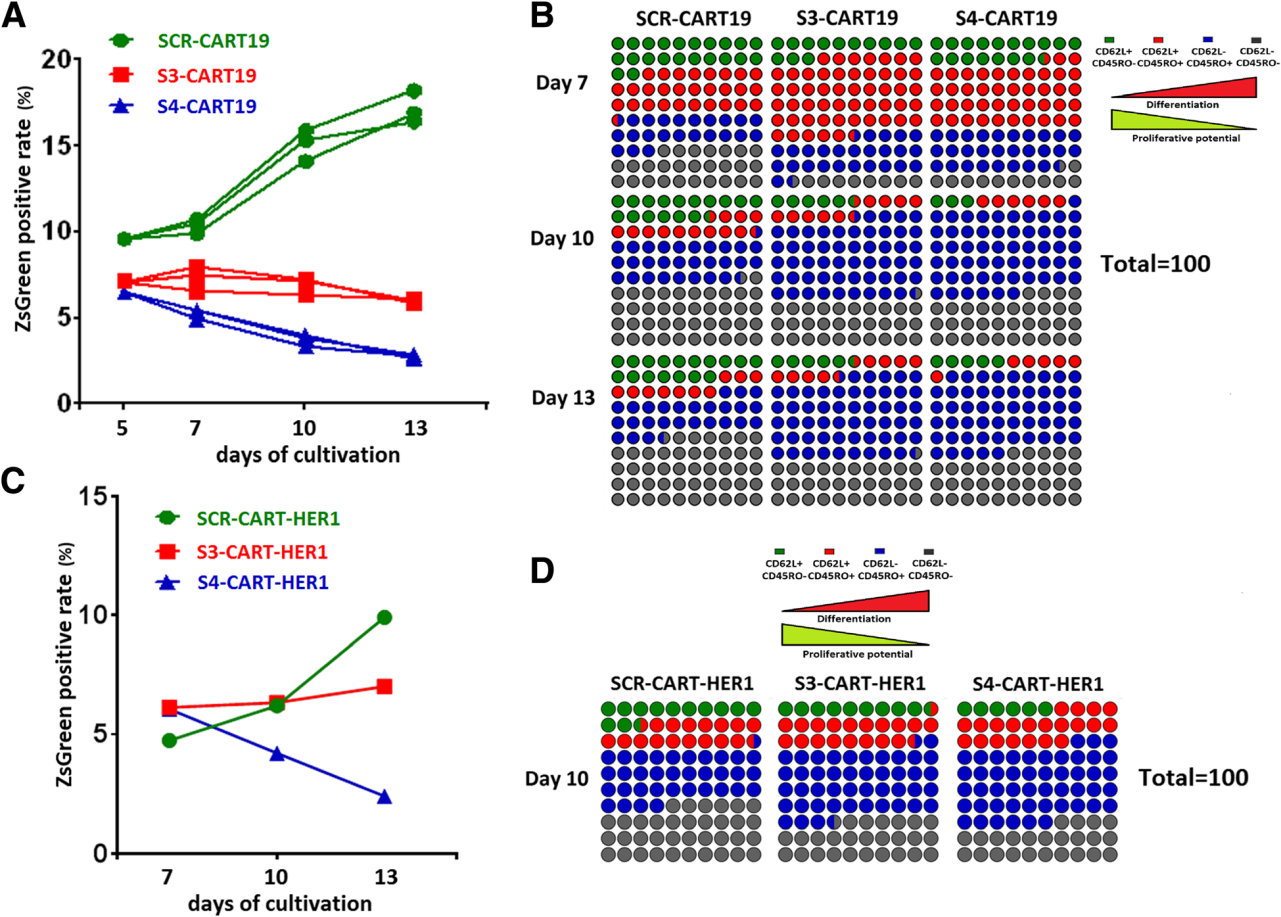
The effect of PD-1 silencing was tested under different culture conditions and in CART-HER1 system. **a** and **b** IL-7, IL-15 and IL-21 were used instead of IL-2 for the cultivation of CART-19 cells. During the cultivation, the ZsGreen positive rates (**a**) and differentiation phenotypes (**b**) of different CAR-T cells were monitored. **c** and **d** The influence of PD-1 knockdown on CART-HER1 cells was evaluated. The ZsGreen positive rates during cultivation (**c**) and differentiation phenotypes on the tenth day of culture (**d**) in different CAR-T cells were monitored. These results indicated that the effect of PD-1 knockdown on CAR-T cells was universal. Data are plotted as individual points

It has been reported that cytokines such as IL-7, IL-15 and IL-21 can retard T cell senescence, and promote differentiation into memory phenotype and proliferation [28–30]. We confirmed that the combined use of these cytokines did enhance the proliferative activity, but the proliferation inhibition caused by PD-1 knockdown could not be rescued (Fig. 7a). Meanwhile, the alteration of differentiation kinetics caused by PD-1 knockdown was still present here (Fig. 7b).

It has been reported that T cells exhaustion could be triggered by unique conformation of CAR19 molecule mediated autonomous activation [31]. To verify whether the impairment of proliferation caused by PD-1 knockdown was also present in other CAR-T systems, we constructed and tested epidermal growth factor receptor (HER-1) targeting CART (SCR-CART-HER1, S3-CART-HER1 and S4-CART-HER1) cells. As shown in Fig. 7c and d, the proliferation potential of CART-HER1 cells was also inhibited by intrinsic PD-1 blockade, and the alteration of phenotypes was similar to that observed in CART-19 cells.

## Discussion

We wondered whether PD-1 blockade is monotonously beneficial to T cells, especially when the blockade is long-lasting. To prove this point, we constructed dual-promoter vectors that could ensure a clear study of the relationship between PD-1 blockade and CAR-T functions. With this system, we demonstrated that PD­1 knockdown would impair the anti-tumor potential and proliferation of CAR-T cells. During in vitro cultivation, the PD-1 silencing altered the differentiation kinetics, and the persistence of CAR-T cells was also attenuated. These findings suggested that PD-1 might not be unfavorable for T cells function invariably. Moreover, it might be essential for maintaining normal proliferative activity and anti-tumor function. This result will inspire us to re-recognize the function of PD-1 and have certain significance for guiding the clinical use of PD-1 blocking therapies.

Previously, several articles have practiced the strategy of intrinsic blockade of PD-1 in T cells with CRISPR/Cas9 or shRNA technologies [17, 32–34]. Their conclusions were inconsistent with ours. In fact, our colleagues in another group had also confirmed that the anti-tumor function of CAR-T cells would be improved by PD-1 knockout. Therefore, we carried out a clinical trial using PD-1 knockout mesothelin-CAR-T cells (NCT03747965).

We speculate that this discrepancy may be caused by the following reasons. First, differences in the intensity of PD-L1 expression may cause bias. If PD-L1 is constantly over-expressed, low levels of PD-1 expression (by knockdown) may still have a sustained and significant inhibitory effect. And we have also observed such a hint (Fig. 2b and Additional file 1: Figure S5C and D),

Second, the PD-1 receptor has two different signaling motifs, immunoreceptor tyrosine-based inhibitory motif (ITIM) and immunoreceptor tyrosine-based switch motif (ITSM). However, whether these motifs work cooperatively or independently is still unknown. In addition, the current evidence suggests that only ITSM but not ITIM is involved in the recruitment of downstream inhibitory molecules, even though both of them are phosphorylated upon activation [35]. Does PD-1 have other functions rather than inhibition? Can it function independently of ligands? In fact, these very reasonable assumptions have been ever proposed or partially verified [23]. We speculate that the knockdown of PD-1 might break its balance of normal functions, and the residual PD-1 receptors may still exert specific regulatory signaling. We believe that this may be the main reasons for the inconsistency between PD-1 knockout and knockout. Although knockdown and knockout are often used to verify each other, the results observed by these two methods sometimes still lead to inconsistent conclusions [36].

Although there was ample evidence that PD-1 knockdown inhibited T cell proliferation, S3-CART19 cells still exhibited a similar in vivo proliferative capacity as SCR-CART19 cells. We thought that this inconsistency might be due to the experimental bias. In the S3-CART19 treatment group, two mice developed abnormally CAR-T amplification than the other samples (Fig. 6d). This bias could be caused by differences in the initial tumor burdens and the administrated CAR-T cell numbers. In addition to proliferation, we also observed that PD-1 knockdown altered the differentiation of CAR-T cells. It seemed that PD-1 knockdown accelerated the early differentiation but prevented effector T cells from entering into effector memory T cells. We speculate that this may be the cause of impaired proliferation of CAR-T cells. Because the killing activity of late-differentiated T cells is more robust, the slightly weaker TCIIR presented by PD-1 knockdown CAR-T cells may be due to the changes in T cell composition (lower proportion of CD62L^−^CD45RO^−^ T cells).

Compared with IL-2, the combination of IL-7, IL-15 and IL-21 significantly increased not only the proportion of early-differentiated T cells, but also the proportion of late-differentiated T cells. Considering that these factors can help T cells maintain a younger status and promotes T cell proliferation activity, we speculate that the early differentiation of T cells is more influenced by signaling, and the late differentiation is more dependent on the cell divisions.

Although we have demonstrated that PD-1 knockdown inhibited the anti-tumor function of CAR-T cells, we still do not insist that the clinical application of PD-1 blockade is discouraging. First, the activation of endogenous CTLs depends on the interaction of T-cell receptor (TCR) and human leukocyte antigen (HLA) complexes and co-stimulatory molecules, and this natural activation process is normally not as abundant as the artificial one. In addition, the immune response induced by natural TCR-HLA interaction is usually milder than that mediated by CAR molecule [37–39]. Although we have reached the same conclusion with different culture protocols and CAR-T systems, we would like to emphasize that this may not apply to all long-lasing PD-1 blockade scenarios, because long-lasting PD-1 blockade are likely to develop different influences on T cells in different statuses [22, 40].

Second, the difference between intrinsic and antibody-dependent PD-1 blockade is obvious. This also suggested that PD-1 might directly regulate T cells but not rely on the involvement of PD-L1. In this scenario, the long-lasting PD-1 blockade by antibody would not perturb the innate function of PD-1, and the antibody drugs would be still promising.

Third, the effect of PD-1 blockade on improving T cell resistance to immunosuppression is undoubted. If the key mechanism by which PD-1 knockdown impairs T cell proliferation could be validated, other strategies could be combined to rescue the adverse effects. And this is the subject we will focus on next.

## Conclusions

Whether PD-1 blockade is monotonously beneficial to T cells remains controversial, especially when the blockade is long-lasting. To improve our understanding of long-lasting PD-1 blockade, we constructed dual-promoter vectors in which PD-1 blockade and CAR molecule expression could be achieved within individual cells to guarantee a clear study of the relationship between PD-1 blockade and T cell functions. With this system, we demonstrated that PD­1 knockdown would impair the in vitro and in vivo anti-tumor potential and proliferation of CAR-T cells. During in vitro cultivation, the PD-1 silencing altered the differentiation kinetics. The persistence of CAR-T cells was also attenuated by PD-1 silencing. These findings suggested that PD-1 signaling might not be unfavorable for T cells function invariably. Moreover, it might be essential for maintaining normal proliferative activity and anti-tumor function. This result will inspire us to re-recognize the function of PD-1 and have certain significance for guiding the clinical use of PD-1 blocking therapies.

## Additional file

Additional file 1 **Figure S1.** Confirmation of the function of the dual promoter vectors. **Figure S2.** Expression of PD-L1 in different tumor cells. **Figure S3.** In vivo expression of CAR molecules and PD-1, tumor burdens and in vivo expansion of CAR-T cells. **Figure S4.** Analysis of in vitro proliferative potential of CAR-T cells. **Figure S5.** The effect of PD-1 knockdown on CAR-T cell function and differentiation status. **Figure S6.** The expression of several potential off-targeted genes. **Figure S7.** PD-1 blocking antibodies barely affect the phenotype and proliferation of CAR-T cells. (DOCX 3914 kb)

## Data Availability

All data generated or analyzed in this study are included in this article and its additional files. Other data that are relevant to this article are available from the corresponding author upon reasonable request.

## Abbreviations

BLI: Bioluminescence imaging
CAR-T: Chimeric antigen receptor modified T
CTL: Cytotoxic T lymphocytes
DST: Double stimulation
E:T: Effector cell to target cell
EF1-α: Elongation factor 1-alpha
HPD: Hyperprogressive disease
LAG-3: Lymphocyte activation gene-3
PBMC: Peripheral blood mononuclear cells
PD-1: Programmed death-1
PD-L1: Programmed death-ligand 1
qRT-PCR: Quantitative real-time PCR
S3: shRNA-3
S4: shRNA-4
SCR: Scramble sequence
shRNA: Short hairpin RNA
SST: Single stimulation
TCIIR: Target cell-induced immune response
TIM-3: Mucin-domain containing-3
WB: Western blotting
