# Supplementary material for: PD-1 silencing impairs the anti-tumor function of chimeric antigen receptor modified T cells by inhibiting proliferation activity
Source: J Immunother Cancer. 2019 Aug 7;7:209. doi: 10.1186/s40425-019-0685-y (PMC6686487; doi:10.1186/s40425-019-0685-y)
Supplement: Supplementary file 1 — Figure S1. Confirmation of the function of the dual promoter vectors. Figure S2. Expression of PD-L1 in different tumor cells. Figure S3. In vivo expression of CAR molecules and PD-1, tumor burdens and in vivo expansion of CAR-T cells. Figure S4. Analysis of in vitro proliferative potential of CAR-T cells. Figure S5. The effect of PD-1 knockdown on CAR-T cell function and differentiation status. Figure S6. The expression of several potential off-targeted genes. Figure S7. PD-1 blocking antibodies barely affect the phenotype and proliferation of CAR-T cells. (DOCX 3914 kb) [file 40425_2019_685_MOESM1_ESM.docx]

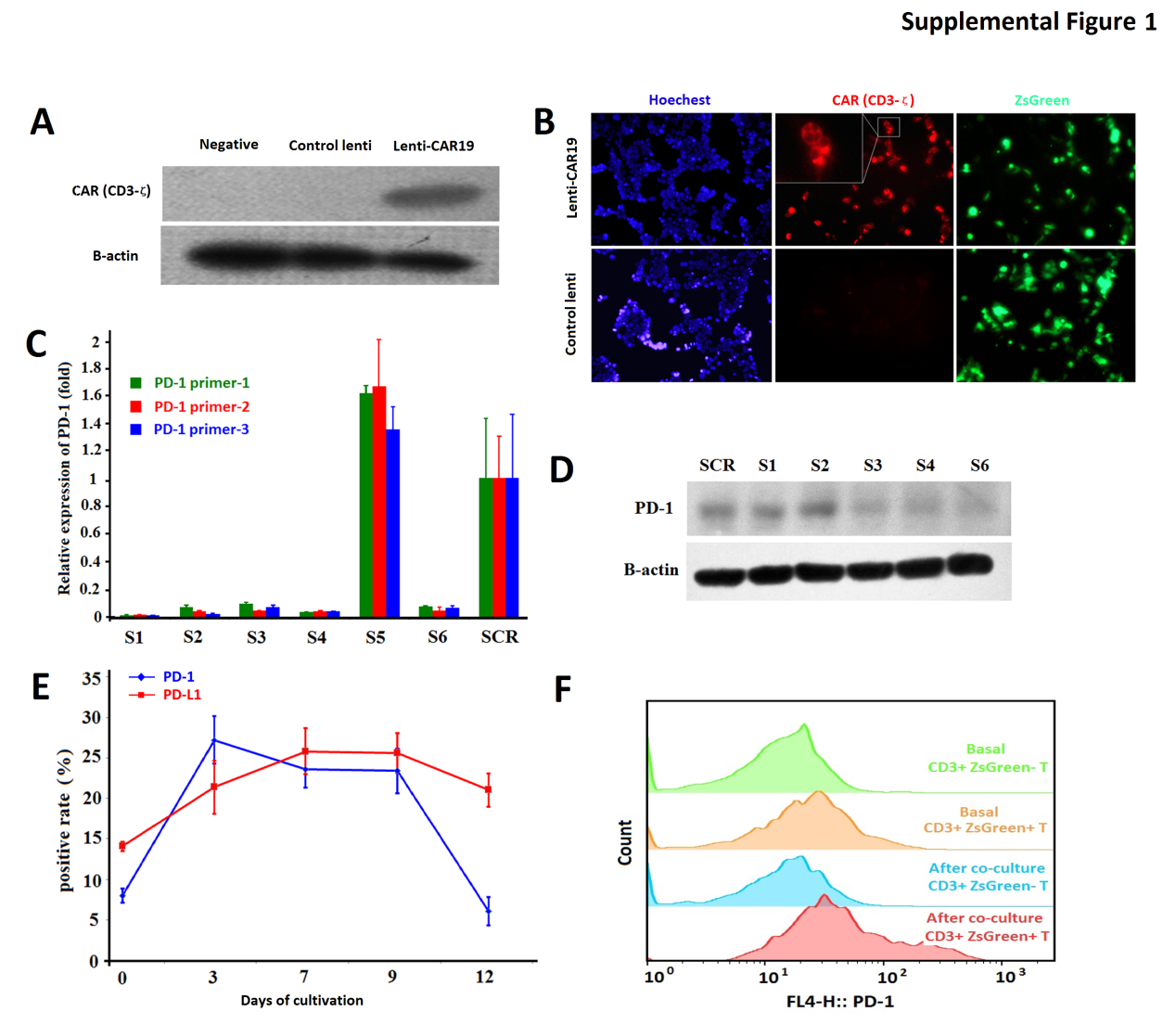


**Figure S1. (A)** The 293T cells were infected with control and constructed lentivirus at multiplicity of infection at 5 (MOI=5). 30ug of total protein was used for the detection of CAR molecule expression. **(B)** The infected 293T cells were subjected to immunofluorescent staining. The same lentivirus without CAR molecule was used as control. The hoechest (blue), CD3-ζ(red) and ZsGreen (green) were detected. **(C and D)** The silencing efficiency of different shRNA sequences in ZsGreen positive sorted Jurkat cells were analyzed by qRT-PCR with three different PD-1 targeting primers **(C)** and western blotting **(D),** and shRNA-5, shRNA-1 and shRNA-2 were excluded. **(E)** The positive rates of PD-1 (blue) and PD-L1 (red) in T cells during the in vitro cultivation were examined by flow cytometry. **(F)** The expression of PD-1 in SCR-CART19 population were measured before and after co-culture with Raji cells. The ZsGreen positive (CAR-T) and ZsGreen negative (non-infected T) cells in the same cell population were analyzed separately, and up-regulation of PD-1 was proved to be CAR-dependent. Data represent the mean ± SEM of triplicates and are representative of at least 3 independent experiments or are plotted as individual points.


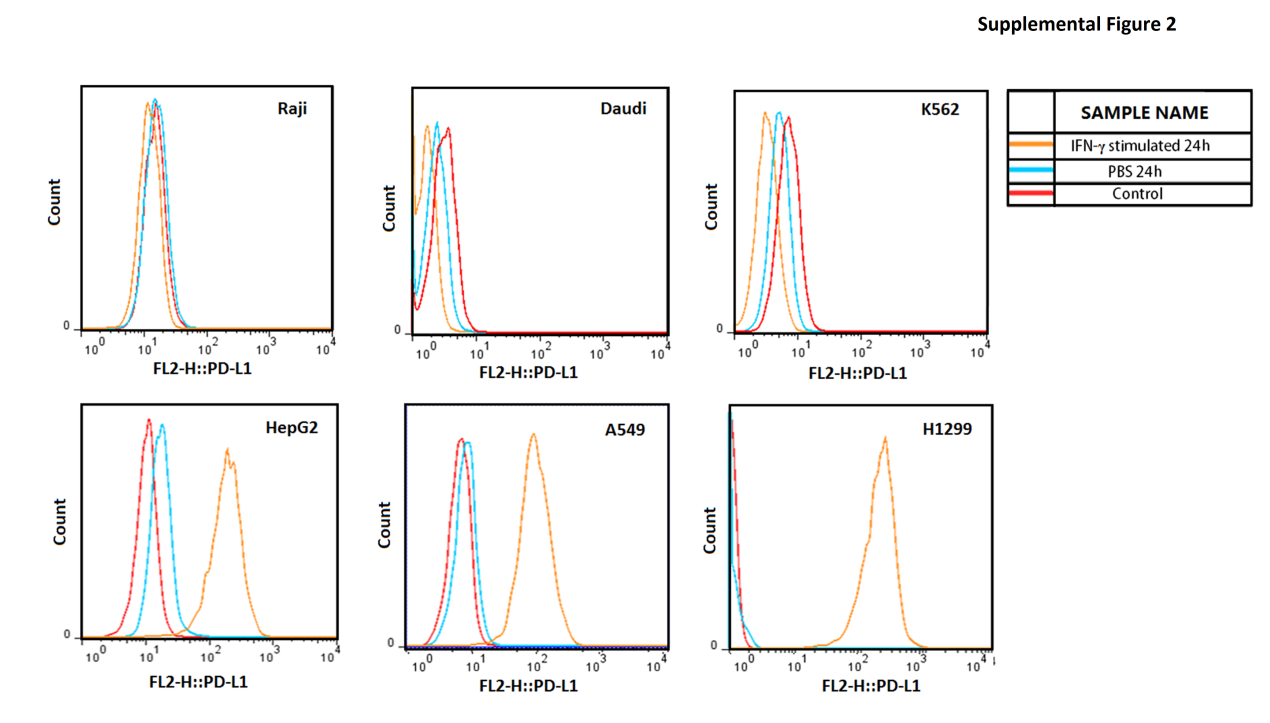


**Figure S2.** The expression of PD-L1 in different tumor cells under normal conditions (red), IFN-γ stimulation (yellow) and PBS control (blue) were tested by flow cytometry. Data are presented individually without replication.


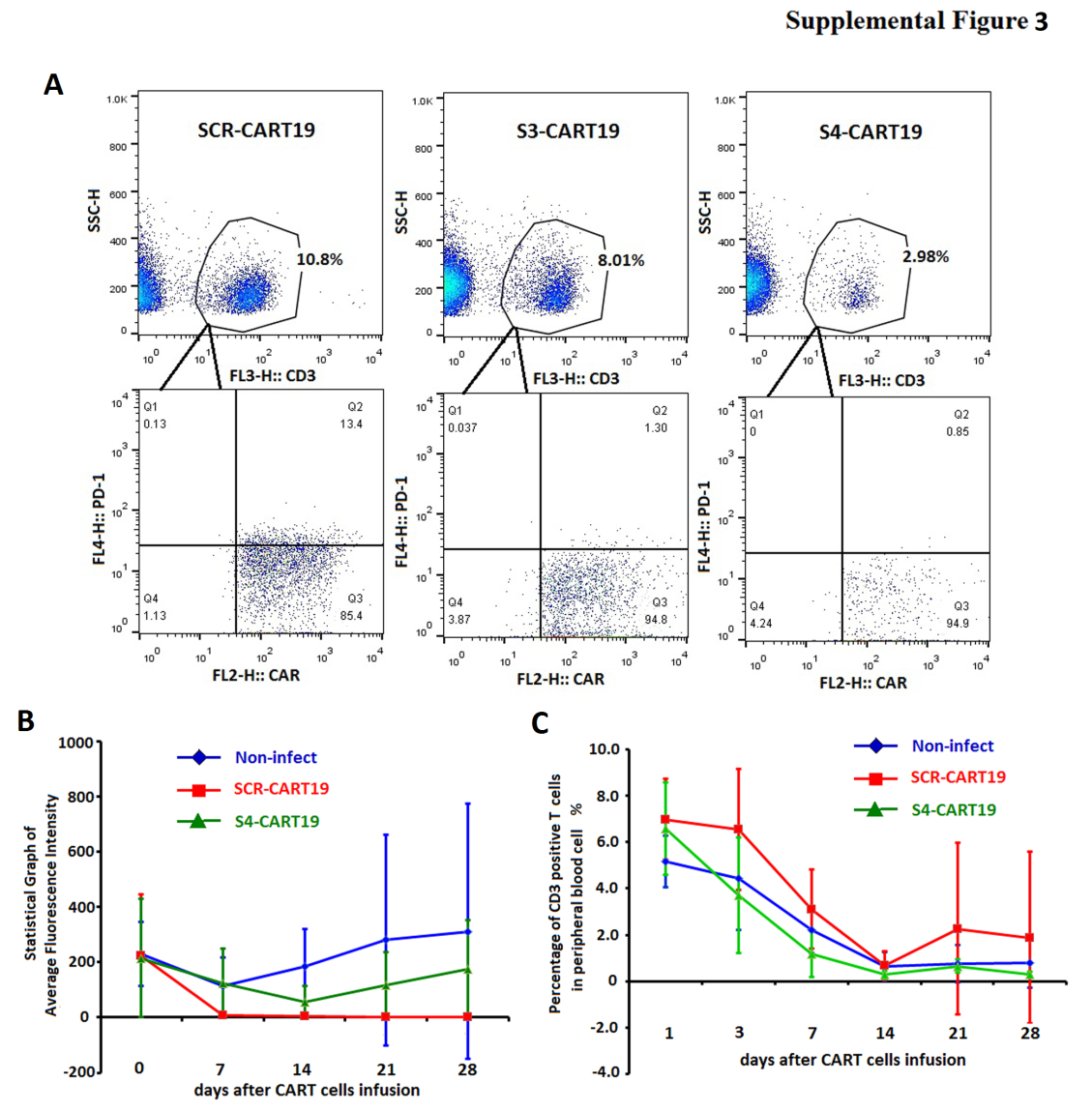


**Figure S3.** **(A)** One week after the reinfusion of CAR-T (1×10^7^ per mouse), we detected the expression of CAR and PD-1 in vivo. Data are presented individually without replication. Statistical data of Figure 3B **(B)** and Figure 3C **(C)**, data represent the mean of six samples in each group.


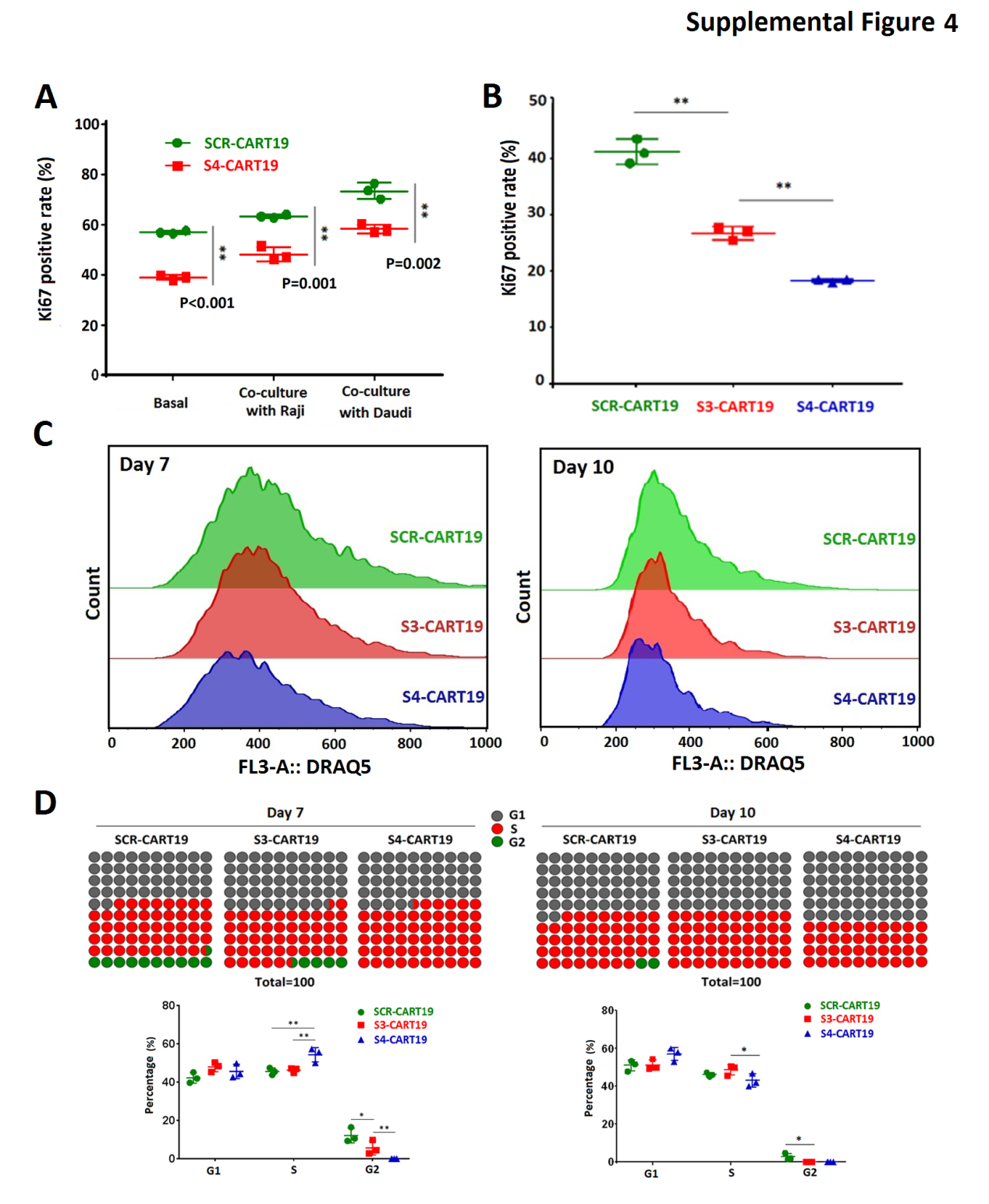


**Figure S4.** **(A)** The Ki67 positive rates in CAR-T cells which were cultured for 7 days were measured before and after target cells stimulation. **(B)** Another PD-1 shRNA sequence, S3, was tested to eliminate the possible off-target effects of S4. The Ki67 positive rates in different CAR-T cells which were cultured for 14 days were presented. The results showed that PD-1 knockdown significantly reduced the positive rate of Ki67 in CAR-T cells. The DRAQ5 staining **(C)** and cell cycle analysis **(D)** were performed in CAR-T cells those were cultured for seven days and ten days. In PD-1 silenced CAR-T cells, the proportion of cells in the division phase was significantly reduced. 0.01<P*<0.05; P**<0.01. Data represent the mean ± SEM of triplicates and are representative of at least 3 independent experiments or are plotted as individual points. Statistical significance was determined using the ANOVA method for multiple comparisons.


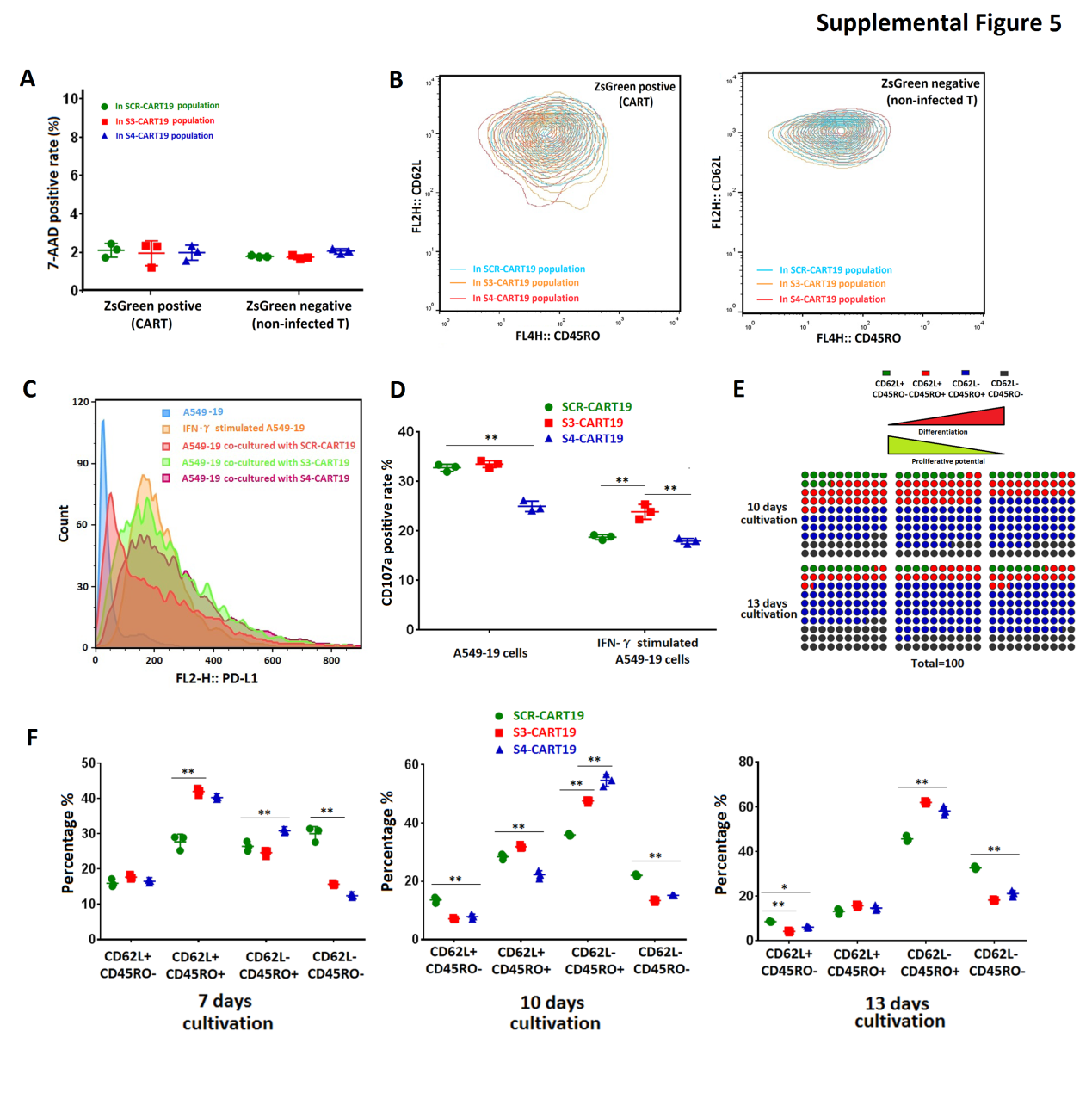


**Figure S5. (A)** 7-AAD staining was performed on CAR-T cells those were cultured for 7 days, and no difference on apoptosis was demonstrated between different CAR-T cells. **(B)** Analysis of the differentiation status of CAR-T cells (left) and their corresponding non-infected T cells (right) in the same cell populations, after ten days cultivation, were presented. **(C)** 24 hours co-culture with CAR-T cells could up-regulate the PD-L1 expression in A549-19 cells, and 50 ng/ml IFN-γ, for 24 hours, could induce a similar PD-L1 expression level. **(D)** With this milder level of PD-L1 expression, we tested different CAR-T cells’ CD107a expression. It was demonstrated that PD-1 knockdown could increase the resistance to PD-L1-mediated immunosuppression, but the residual PD-1 in S3-CART19 and S4-CART19 cells still significantly inhibited the expression of CD107a. **(E and F)** Statistical graphs of the proportion of different phenotypes at different time points. 0.01<P*<0.05; P**<0.01. Data represent the mean ± SEM of triplicates and are representative of at least 3 independent experiments or are plotted as individual points. Statistical significance was determined using the ANOVA method for multiple comparisons.


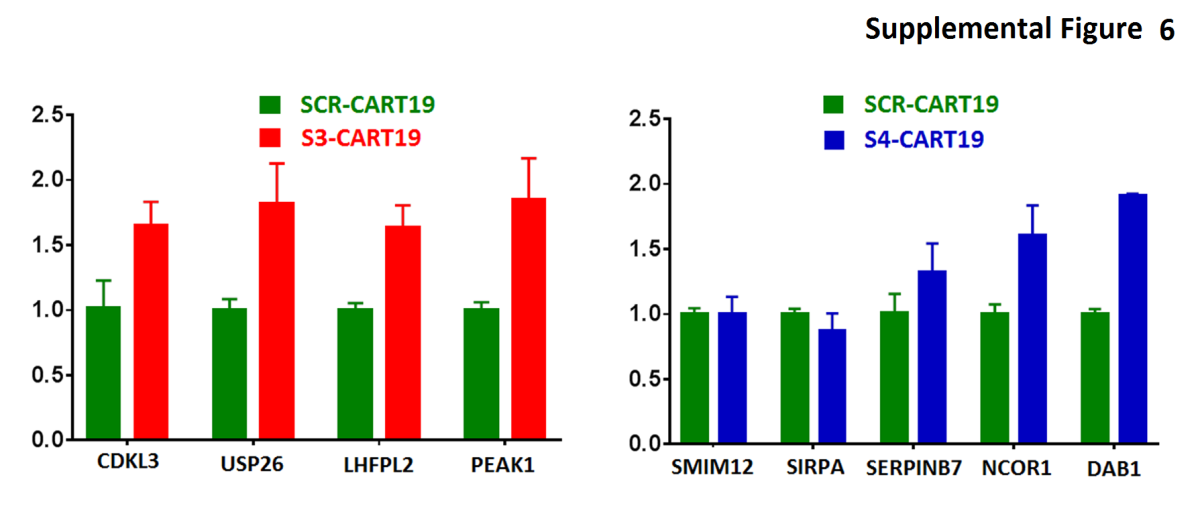


**Figure S6.** Several genes most likely to be mistargeted by S3 or S4 were detected by qRT-PCR to further exclude the possibility of off-target


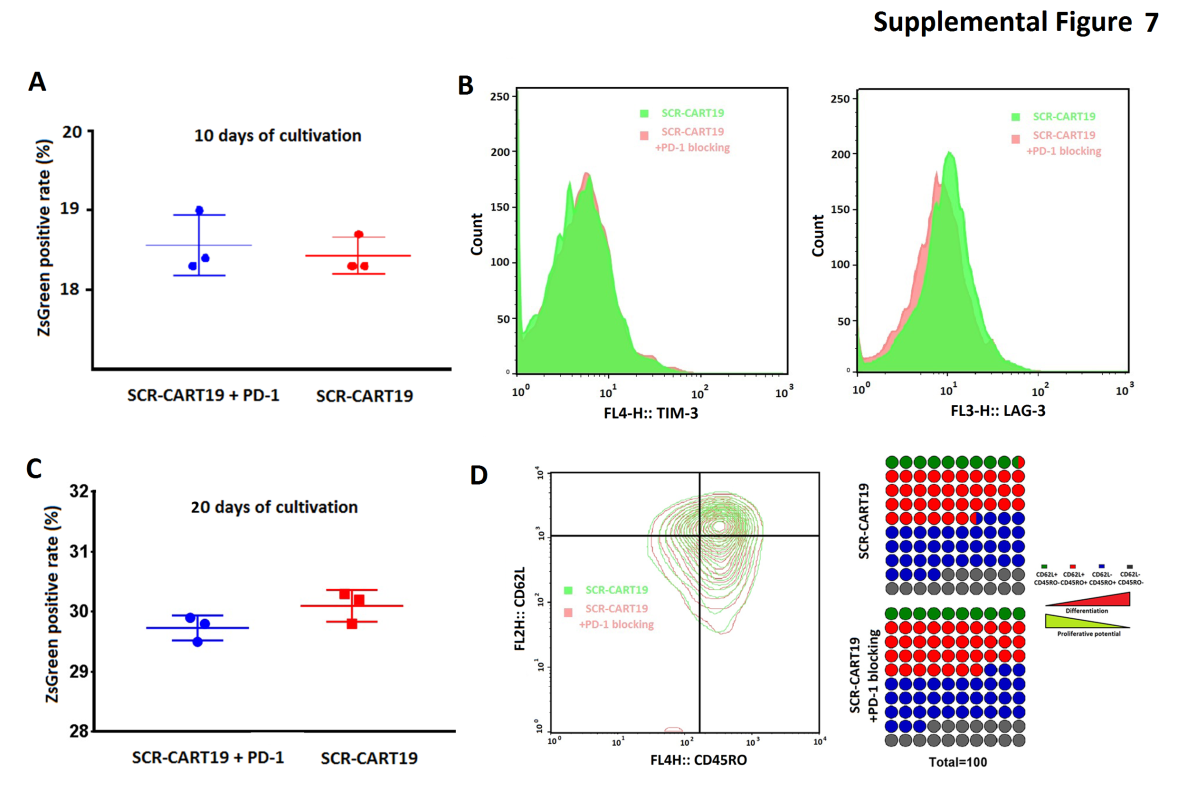


**Figure S7. (A)** The ZsGreen positive rates in PD-1 antibody added and normally cultured SCR-CART19 cells those were cultured for ten days were presented. **(B)** The expression of TIM-3 and LAG-3 in PD-1 antibody added and normally cultured SCR-CART19 cells those were cultured for twenty days were detected. **(C)** The ZsGreen positive rates in PD-1 antibody added and normally cultured SCR-CART19 cells those were cultured for twenty days were presented. **(D)** The phenotype of PD-1 antibody added and normally cultured SCR-CART19 cells those were cultured for twenty days were presented. Together, these results demonstrated that the PD-1 blockade by antibodies exerted little effect on CAR-T cells. Statistical significance was determined using ANOVA method for multiple comparisons. Data represent the mean ± SEM of triplicates and are representative of at least 3 independent experiments or are plotted as individual points.
